# Comparative Analysis of Perfusion Index and End-Tidal Carbon Dioxide in Cardiac Arrest Patients: Implications for Hemodynamic Monitoring and Resuscitation Outcomes

**DOI:** 10.7759/cureus.50818

**Published:** 2023-12-20

**Authors:** Baki Dogan, Emre Kudu, Faruk Danış, Elif Ozturk Ince, Mehmet A Karaca, Bulent Erbil

**Affiliations:** 1 Emergency Medicine, Medical Point Gaziantep Hospital, Gaziantep, TUR; 2 Emergency Medicine, Marmara University Pendik Training and Research Hospital, Istanbul, TUR; 3 Emergency Medicine, Bolu Izzet Baysal Training and Research Hospital, Bolu, TUR; 4 Emergency Medicine, Hacettepe University Hospital, Ankara, TUR

**Keywords:** resuscitation, photoplethysmography, perfusion index, outcome, end-tidal carbon dioxide, cardiac arrest, advanced cardiac life support

## Abstract

Background: During cardiopulmonary resuscitation (CPR), some parameters (e.g., intraarterial pressure measurement and end-tidal carbon dioxide (EtCO_2_)) indicate the quality and outcome of resuscitation. These parameters are generally based on monitoring the hemodynamic status. Perfusion index (PI) is a calculation from the photoplethysmography (PPG) signal and displays the proportion of pulsatile to non-pulsatile light absorption or reflection in the PPG signal. It helps to evaluate cardiac output and tissue perfusion in the care of a critical patient. Its most important advantages are that it can be easily measured with a pulse oximeter probe attached to the finger (non-invasive), can be objectively repeated, can be applied quickly, and is inexpensive. Normal PI values range from 0.2% to 20%. Despite being recognized as a valuable indicator of hemodynamics, there is limited information regarding its relevance in patients experiencing cardiac arrest. Although the PI is known to be a valuable parameter to indicate hemodynamics, information about its value in cardiac arrest patients is limited. This study aims to evaluate the performance of PI and EtCO_2_ in predicting the return of spontaneous circulation (ROSC) among cardiac arrest patients.

Methods: This was a single-center, prospective, observational clinical study including both out-of-hospital and in-hospital adult cardiac arrest patients. The study was conducted from November 1, 2018 to April 30, 2019 at the Emergency Department (ED) of the Hacettepe University Hospital, Ankara, Turkey. The EtCO_2 _values of the patients were recorded at the time they were intubated (t_0_) and every five minutes (t_5_, t_10_, t_15_...) during CPR. Along with these measurements, PI values were measured with the Masimo Signal Extraction Technology device (Masimo, California, United States). The study's primary outcome was PI's performance in predicting the ROSC among cardiac arrest patients. The secondary outcomes of the study were the performance of EtCO_2_ in predicting the ROSC among cardiac arrest patients and the association between PI and EtCO_2_ values.

Results: We included a total of 100 cases. The mean age of patients was 70.4 ± 13.4 years, and 65% were male. The ROSC was achieved in 29 patients. There was no statistical difference in PI values between the ROSC (+) and ROSC (-) groups at any minute. However, in the ROSC (+) group, EtCO_2_ values were observed to be high starting from the fifth minute (t_5_, p=0.010; t_10_, p<0.001; t_15_, p=0.014; t_20_, p=0.033; t_25_, p=0.003, respectively). There was no correlation between the PI and EtCO_2_ values at 0, 5, 10, 15, 20, and 25 minutes (t_0_, p=0.436; t_5_, p=0.154; t_10_, p=0.557; t_15_, p=0.740; t_20_ p=0.241; t_25_ p=0.201, respectively).

Conclusion: Measuring PI values during resuscitation in intubated cardiac arrest patients does not help clinicians predict the outcome. In addition, no correlation was found with EtCO_2_ values. However, EtCO_2_ values remained high in patients with the ROSC from the fifth minute onward. Further larger-scale studies are needed regarding the optimal use of PI in cardiac arrest patients.

## Introduction

Cardiac arrest is one of the leading causes of deaths worldwide [1,2]. Even with appropriate resuscitation of the patient, hospital survival and discharge rates with good neurological outcomes remain low [1,3,4]. According to the data published by the American Heart Association (AHA) in 2022, the hospital discharge rate is 9.0% for out-of-hospital cardiac arrests (OHCAs), while this rate is 23.3% for in-hospital cardiac arrests (IHCAs) [1].

During cardiopulmonary resuscitation (CPR), some parameters show the effectiveness of CPR and predict the likely outcome of resuscitation [1]. These parameters are generally based on monitoring the hemodynamic status, such as intraarterial pressure measurement and end-tidal carbon dioxide (EtCO_2_) [1,3]. EtCO_2_ represents the level of carbon dioxide that is released at the end of an expiration. Studies have shown that this value correlates with blood flow and cardiac output during CPR, provides information about the quality of CPR, and predicts the potential outcome [3,5,6]. Low EtCO_2_ values (<10 mmHg) indicate esophageal intubation or poor quality of CPR, while sudden increases in EtCO_2_ values overreaching 10 mmHg that remain higher than preceding values may indicate the return of spontaneous circulation (ROSC) [7].

Photoplethysmography (PPG) is another monitoring technique and has long been widely used in many fields of medicine, including hemodynamic monitoring in the form of pulse oximetry [8,9]. The perfusion index (PI) is a calculation used for hemodynamic monitoring. It is estimated from the PPG signal and displays the proportion of pulsatile to non-pulsatile light absorption or reflection in the PPG signal [8,10]. It helps to evaluate cardiac output and tissue perfusion in critical patients. Normal PI values range from 0.2% to 20% [11]. Its most important advantages are that it can be easily measured with a pulse oximeter probe attached to the finger (non-invasive), can be objectively repeated, can be applied quickly, and is inexpensive [12,13]. Understanding PPG and PI's fundamental principles and limitations is crucial for correctly using it and getting accurate results [10]. Although the PI is known to be a valuable parameter to indicate hemodynamics, information about its value in cardiac arrest patients is limited [14]. This study aims to evaluate the performance of PI and EtCO_2_ in predicting the ROSC among cardiac arrest patients.

## Materials and methods

Study settings

This was a single-center, prospective, observational clinical study including both adult OHCA and IHCA patients. The study was conducted from November 1, 2018 to April 30, 2019 at the Emergency Department (ED) of the Hacettepe University Hospital, Ankara, Turkey. The hospital is a tertiary care center affiliated with a medical school accommodating an annual volume of approximately 100,000 adult patient presentations to the ED. This study was reported in accordance with the Strengthening the Reporting of Observational Studies in Epidemiology (STROBE) guidelines [15]. Hacettepe University Clinical Research Ethics Committee approved the study protocol (protocol number: GO 18/620-37).

Study population

All cardiac arrest patients over the age of 18 years who presented to the ED or who collapsed in the ED (IHCAs) were included consecutively in the study. Informed consent was obtained from the patient's relatives for participation in the study. Since EtCO_2_ values could not be measured in non-intubated patients, patients who were not intubated within the first 10 minutes of resuscitation were excluded. Patients who withdrew consent or had incomplete data were also excluded from the study. The patients were divided into groups according to the ROSC.

Study design and protocol

Hacettepe University Hospital Emergency Department implements the advanced cardiac life support protocol (ACLS) according to the AHA and International Liaison Committee on Resuscitation (ILCOR) recommendations [16,17]. The ACLS protocol applied to out-of-hospital and in-hospital cardiac arrest patients is essentially the same, although it contains minor changes [16]. While activation of emergency response comes to the fore in OHCA cases, early recognition and prevention come to the fore in IHCA cases. High-quality CPR and defibrillation are the main elements in both resuscitations [16].

When an OHCA case was presented to the hospital or an IHCA case was recognized, high-quality CPR was initiated quickly following the guidelines [16,17]. The patient was monitored with the LIFEPAK® 15 monitor/defibrillator device (Physio-Control, Redmond, Washington, USA), and the cardiac rhythm was monitored. Oximetric measurements (saturation and PI) were made from the fingertip with the Masimo Signal Extraction Technology (SET®, Masimo, California, United States) Radical 7 pulse device. Resuscitation guidelines emphasize that effective bag mask valve application will be as effective as an advanced airway application [16]. Therefore, the primary physician decided the ventilation method to be applied to the patient [16,18]. If the patient's primary physician deemed it necessary, advanced airway application (mostly endotracheal intubation) was provided. The most experienced physician in the airway provided the intubation process [16], and then the medical staff attached an EtCO_^2^_ detector to the monitor (LIFEPAK® 15 monitor/defibrillator) via an endotracheal tube. According to the AHA's current guidelines, it is stated that EtCO_2_ values may not reflect the real value in non-intubated patients [16]. Therefore, EtCO_2_ monitoring is not performed in patients who were not intubated. Resuscitation continued until the ROSC was achieved, or the team leader decided to terminate resuscitation. The ROSC was defined as a palpable pulse from the carotid artery [3].

Variables and data measurement

The prehospital data of patients were collected from emergency medicine system charts and patients' relatives or direct providers. The demographics and hospital course were obtained from the hospital’s electronic system record. Neurological outcomes of surviving patients were evaluated using the cerebral performance category scale (CPC 1-5) score on the 30th day. While hospitalized patients were examined directly, discharged patients or their relatives were reached by phone, and their conditions were evaluated. A favorable neurological outcome is defined as a CPC ≤2.

With intubation of patients, EtCO_2_ values began to be measured with the LIFEPAK® 15 monitor/defibrillator. The EtCO_2_ values of the patients were recorded at the time they were intubated (t_0_) and every five minutes (t_5_, t_10_, t_15_, t_20_, t_25_...) as long as resuscitation continued. PI values could be measured from the beginning of resuscitation with the Masimo Signal Extraction Technology (SET®) Radical 7 pulse oximeter. These values were started to record after intubation with the EtCO_2_ measurement. After intubation, initial PI values were recorded, and afterward, PI values were recorded every five minutes with EtCO_2_.

Outcomes

The study's primary outcome was PI's performance in predicting the ROSC among cardiac arrest patients. The secondary outcomes of the study were the performance of EtCO_2_ in predicting the ROSC among cardiac arrest patients and the association between PI and EtCO_2_ values.

Statistical analysis

The IBM SPSS Statistics for Windows, version 23 (released 2015; IBM Corp., Armonk, New York, United States) and R program (R Foundation for Statistical Computing, Vienna, Austria) was used for the statistical analysis. Shapiro-Wilk test was used to evaluate the distribution of data. Categorical variables are presented as numbers with percentages and numerical variables as medians with interquartile ranges. For comparisons between groups, a chi-squared test was used for categorical variables, and the Mann-Whitney U test was used for numerical variables. Spearman correlation analysis was performed to determine the correlation between two numerical variables. Statistical significance was set at p < 0.05.

## Results

**Figure 1:**
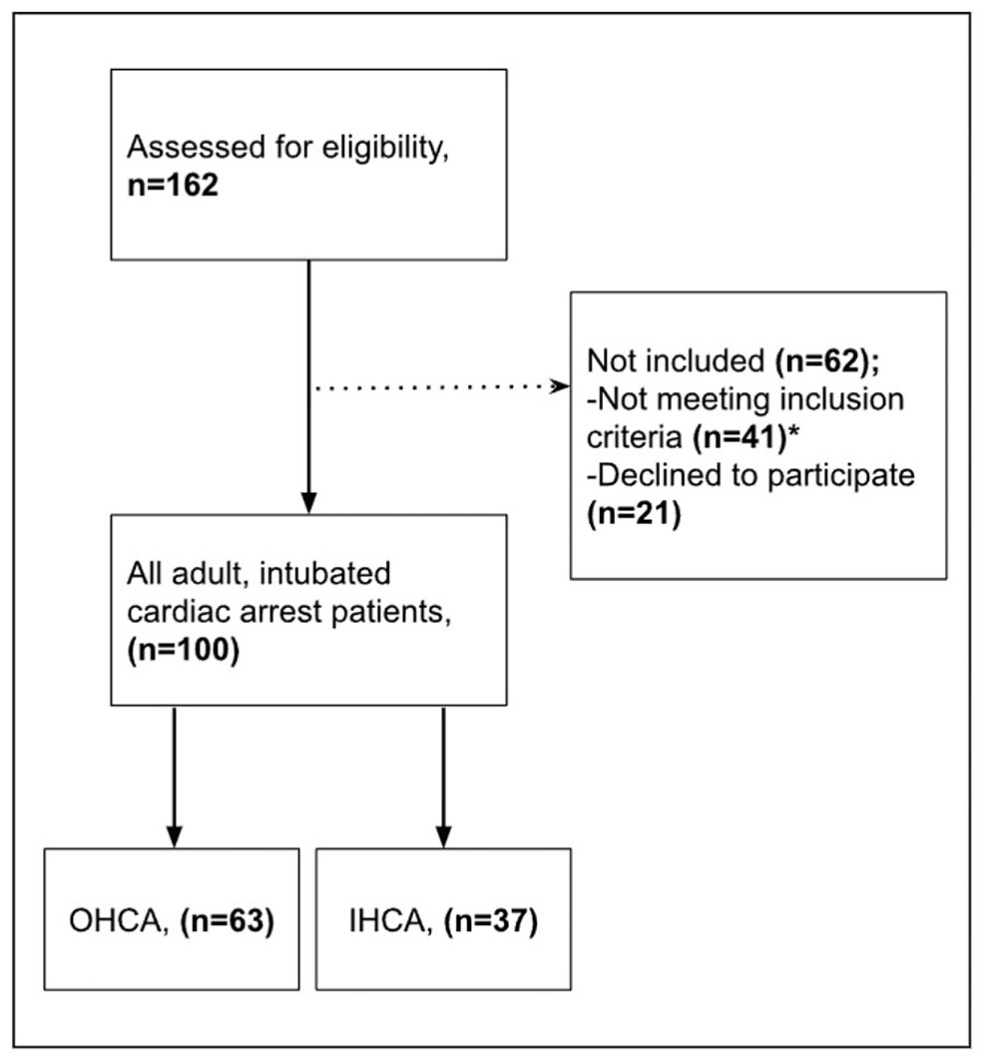
Study flow diagram. *Eight of the patients had the pre-hospital return of spontaneous circulation, 13 of them had delayed intubation (>10 minutes), and 20 of them were not intubated.

During the study, a total of 162 cardiac arrest cases were observed, both IHCAs and OHCAs. Of these, 21 of them were not included in the study because their relatives did not agree to participate in the study, and 41 of them were not included in the study due to missing data (eight of them had pre-hospital ROSC, 13 of them had delayed intubation, and 20 of them were not intubated). As a result, 63 OHCA and 37 IHCA patients were included in the study (Figure 1).

The patient ages ranged from 24 to 94 years, with a mean of 70.4 ± 13.4, and 65% of the patients were male. The ROSC was achieved in 29 (29%) of the patients. The characteristics of patients and cardiac arrest data are given in Table 1.

**Table 1:** Characteristics and outcomes of cardiac arrest patients eligible for the study (n=100) CPC, cerebral perfusion scale; IHCA, in-hospital cardiac arrest; OHCA, out-of-hospital cardiac arrest; ROSC, return of spontaneous circulation; SD, standard deviation

Characteristics	Number (%)
Age (years), mean ± SD	70.4 ± 13.4
Male, n (%)	65 (65.0)
Location of arrest, n (%)	
OHCA	63 (63.0)
IHCA	37 (37.0)
Presumed etiology, n (%)	
Cardiac	47 (47.0)
Respiratory	22 (22.0)
Metabolic	21 (21.0)
Trauma	3 (3.0)
Unknown	7 (7.0)
Shocakble rhythm, n (%)	25 (25.0)
ROSC	29 (29.0)
One-month survival	10 (10.0)
Neurologic outcome, n (%)	
CPC 1 or 2	3 (3.0)
CPC 3 or 4	2 (2.0)
CPC 5	5 (5.0)

The PI and EtCO_2_ values were examined to predict the ROSC. There was no statistical difference in the PI values between groups (Table 2). However, the EtCO_2_ values were significantly higher in the ROSC (+) group than in the ROSC (-) group starting from the fifth minute (Table 3). Comparison and further analysis were not performed due to the significant decrease in sample size after the 25th minute. When the patients were divided into subgroups as IHCAs and OHCAs, no statistical difference was observed in the PI and EtCO_2_ values between patients with and without ROSC (p > 0.05).

**Table 2:** PI values of ROSC (+) and (-) patients by minutes. IQR, interquartiller range; PI, perfusion index; ROSC, return of spontaneous circulation; *Mann-Whitney U.

Time	ROSC (-)		ROSC (+)		
	n	PI, median (IQR)	n	PI, median (IQR)	p*
t0	71	1.04 (0.62-2.52)	29	1.08 (0.63-3.82)	0.445
t5	71	1.29 (0.61-2.76)	21	1.20 (0.66-2.53)	0.669
t10	71	0.98 (0.55-2.38)	18	1.21 (0.45-3.29)	0.510
t15	71	1.54 (0.62-2.72)	9	2.37 (0.96-5.07)	0.190
t20	70	0.68 (0.36-1.49)	6	1.08 (0.57-2.85)	0.187
t25	29	1.04 (0.40-2.06)	4	1.10 (0.66-3.30)	0.439

**Table 3:** EtCO2 values of ROSC (+) and (-) patients by minutes. EtCO_2_, end-tidal carbon dioxide; IQR, interquartiller range; ROSC, return of spontaneous circulation; *Mann- Whitney U.

Time	ROSC (-)		ROSC (+)		
	n	EtCO_2_, median (IQR)	n	EtCO_2_, median (IQR)	p*
t0	71	15.0 (10.0-24.0)	29	19.0 (13.0-27.5)	0.110
t5	71	15.0 (10.0-20.0)	21	18.0 (13.0-29.0)	0.010
t10	71	16.0 (11.0-21.0)	18	29.0 (21.0-38.3)	<0.001
t15	71	19.0 (13.0-24.0)	9	33.0 (18.0-44.0)	0.014
t20	70	18.0 (11.0-21.0)	6	25.5 (16.8-37.0)	0.033
t25	29	16.0 (13.0-23.0)	4	31.0 (26.0-48.8)	0.003

**Figure 2:**
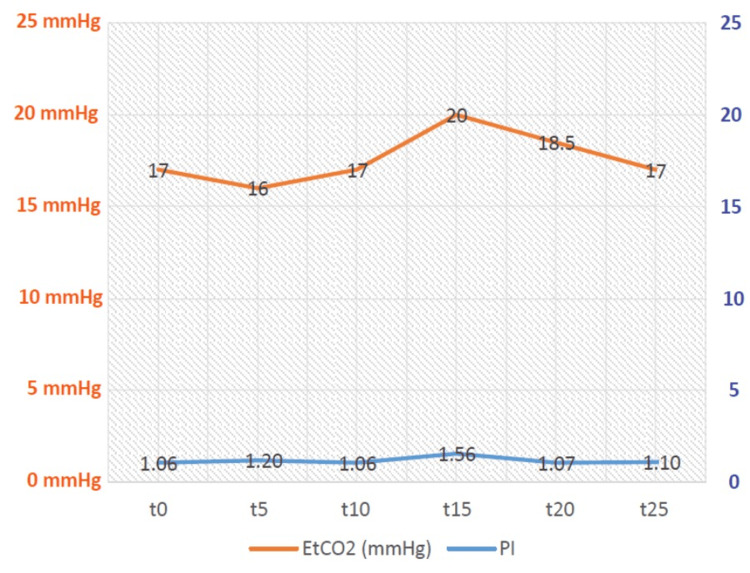
End-tidal carbon dioxide and perfusion index median values over time EtCO_2_, end-tidal carbon dioxide; PI, perfusion index; t, time (minutes)

Spearman correlation analyses were performed to examine the correlation between the EtCO_2_ values and PI. There is no correlation between the PI and ETCO_2_ at 0, 5, 10, 15, 20, and 25 minutes (p=0.436, p=0.154, p=0.557, p=0.740, p=0.241, p=0.201, respectively) (Figure 2).

## Discussion

This study focused on comparing PI and EtCO_2_ in cardiac arrest patients, as these parameters play a potentially important role in hemodynamic monitoring [10,16]. According to our findings, there was no statistically significant difference in the PI values between the groups with and without the ROSC. However, the EtCO_2_ values remained significantly elevated in the ROSC group from the fifth minute onward. This suggests that EtCO_2_ may provide crucial insights into cardiac resuscitation outcomes, whereas the PI cannot be used for the same purpose.

The effectiveness of resuscitation in cardiac arrest patients, its possible consequences, and how long it will continue have always been challenging for physicians [19]. Although many studies have been conducted on this subject, no single parameter gives definitive results about the outcome. Current guidelines recommend that these parameters can guide physicians and be evaluated together [16]. One of the most used parameters for this purpose is EtCO_2_. Our study indicated that EtCO_2_, particularly from the fifth minute onward, was a more prominent indicator for predicting the ROSC. This aligns with established literature supporting EtCO_2_ as a marker reflecting blood flow and cardiac output during resuscitation, predicting the achievement of the ROSC [6]. Therefore, monitoring hemodynamic parameters, especially using EtCO_2_, in the management of cardiac arrest may be critical for evaluating resuscitation effectiveness and determining targeted treatment strategies. On the contrary, PI values did not prove to be determinative in predicting the ROSC. This result suggests that, unlike EtCO_2_, PI might not be as robust a prognostic indicator of cardiac arrest. In addition, when we evaluated with the correlation analysis, it was seen that there was no correlation between EtCO_2_ and PI.

Although normal values of PI are said to be between 0.2% and 20.0% [11], an observational study showed a median (quartiles) standard value of PI of 4.3 (2.9-6.2) [12]. Our study's initial median PI value of cardiac arrest patients was 1.06, which was very low compared to this value. Impaired perfusion of cardiac arrest patients may have caused this situation. Although it is hypothesized that providing quality CPR and ROSC will improve this perfusion [11] and, thus, increase PI values during resuscitation, no difference was detected in patients with and without ROSC. Factors, such as the vasoconstrictor effects of administered adrenaline and the physiological impacts of cardiac arrest, may contribute to delayed perfusion to the extremities during resuscitation [20]. In the study conducted by Savastano et al., they found that the measurement made at the 30th minute in patients with the ROSC was predictive of the patient's 30-day mortality and neurological outcome [12]. Although it does not seem to be a guiding parameter for the physician during resuscitation, PI monitoring may have prognostic value in ROSC patients.

Assessing the limitations of our study is crucial. First, being a single-center study limits the generalizability of our findings. Since EtCO_2_ values only give accurate results in intubated patients, only intubated patients were included in the study. Since non-intubated patients were not included, no results were obtained for this group. However, we predict that EtCO_2_ will become more widespread with developing technology, and further studies will be conducted using appropriate methods on patients who are not intubated. Since Utstein-style reporting is not widely used in Turkey, our prehospital data were limited. Evaluations of these variables could not be made.

## Conclusions

Measuring PI values during resuscitation in intubated cardiac arrest patients does not help clinicians to predict the outcome. In addition, no correlation was found between EtCO_2_ and PI values. However, EtCO_2_ values remained high in patients with ROSC from the fifth minute onward and guided clinicians about the outcome. Further larger-scale studies are needed regarding the optimal use of PI in cardiac arrest patients.
